# Resolution of Visual Hallucinations in Parkinson’s Disease After Right Occipitoparietal Subcortical Hemorrhage: A Case Report

**DOI:** 10.7759/cureus.103306

**Published:** 2026-02-09

**Authors:** Haruo Nishijima, Eri Shibuya, Shota Seino, Youhei Mikami, Masahiko Tomiyama

**Affiliations:** 1 Neurology, Hirosaki University Graduate School of Medicine, Hirosaki, JPN

**Keywords:** false sense of presence, parkinson’s disease, precuneus, subcortical hemorrhage, visual hallucinations

## Abstract

Visual hallucinations are common non-motor symptoms of Parkinson’s disease (PD) that substantially impair quality of life, particularly in advanced stages. However, the neural substrates underlying visual hallucinations in PD remain incompletely understood. Here, we report a rare case of a 72-year-old right-handed man with PD whose visual hallucinations completely resolved after a right occipitoparietal subcortical hemorrhage. He developed PD at 58 years of age and later experienced wearing-off, dyskinesia, cognitive decline, visual hallucinations, and a false sense of presence. At 72 years of age, he presented with headache, nausea, and worsening gait. Neuroimaging demonstrated a right occipitoparietal subcortical hemorrhage. After the hemorrhagic event, visual hallucinations disappeared completely, whereas the false sense of presence persisted. Follow-up MRI three months later showed a residual lesion confined to the right precuneus. The patient remained free of visual hallucinations for several months. This case suggests that the right occipitoparietal region, particularly the precuneus, may play an important role in the generation of visual hallucinations in PD, while persistence of non-visual hallucinations supports partially distinct neural substrates. To our knowledge, this is the first reported case of PD in which visual hallucinations resolved following a hemorrhagic event, providing insight into the neuroanatomical mechanisms of hallucinations in PD.

## Introduction

Parkinson’s disease (PD) is a neurodegenerative disorder characterized by the progressive loss of dopaminergic neurons in the substantia nigra. The cardinal manifestations of PD include motor symptoms such as bradykinesia, tremors, rigidity, and postural instability; however, in recent years, it has been observed that PD is often accompanied by non-motor symptoms in its early stages. These non-motor symptoms may affect a patient’s quality of life to a degree comparable to, or even greater than, that of motor symptoms [1,2].

Visual hallucinations are among the most common non-motor symptoms of PD. Hallucinations often emerge in advanced stages, with visual hallucinations being particularly frequent. Although the mechanisms underlying visual hallucinations in PD are not fully understood, functional and structural abnormalities across multiple brain regions have been implicated [3-5]. For example, Goldman et al. reported that PD patients with visual hallucinations show gray matter atrophy in the cuneus, lingual, and fusiform gyri; middle occipital lobe; inferior parietal lobule; and cingulate, paracentral, and precentral gyri-regions involved in visuoperceptual processing [6].

In addition to visual hallucinations, PD patients may experience other psychiatric symptoms, such as a false sense of presence [7]. However, the neural substrates underlying these non-visual phenomena remain unclear.

Here, we report the case of a patient with PD who experienced a resolution of visual hallucinations following an intracerebral hemorrhage. This case thus provides important insights into the neural origins of visual hallucinations in patients with PD.

## Case presentation

A 72-year-old right-handed man presented to our hospital with complaints of headache, nausea, and worsening gait disturbances. He was diagnosed with PD at 58 years of age, which initially manifested as bradykinesia. Dopamine transporter and 123I-metaiodobenzylguanidine myocardial scintigraphy indicated reduced uptake, consistent with PD. As the disease progressed, the patient developed wearing-off symptoms, dyskinesia, cognitive decline, and visual hallucinations, which became particularly prominent at approximately 71.5 years of age. He began to see several people inside his home, the number of whom subsequently increased. He said that at times students had come to his house to study and that he prepared baths and served meals for them. In addition to these symptoms, he exhibited behavior consistent with person misidentification. For instance, he would look at his wife, fail to recognize her, and ask where his wife had gone. These psychotic symptoms were evaluated by his outpatient clinician, a neurologist, and a movement disorder specialist. His symptoms met the diagnostic criteria for psychosis in PD proposed by the NINDS-NIMH Work Group [8]. At presentation, his medications included levodopa (800 mg/day), selegiline (5 mg/day), opicapone (25 mg/day), and rivastigmine (13.5 mg/day).

**Figure 1:**
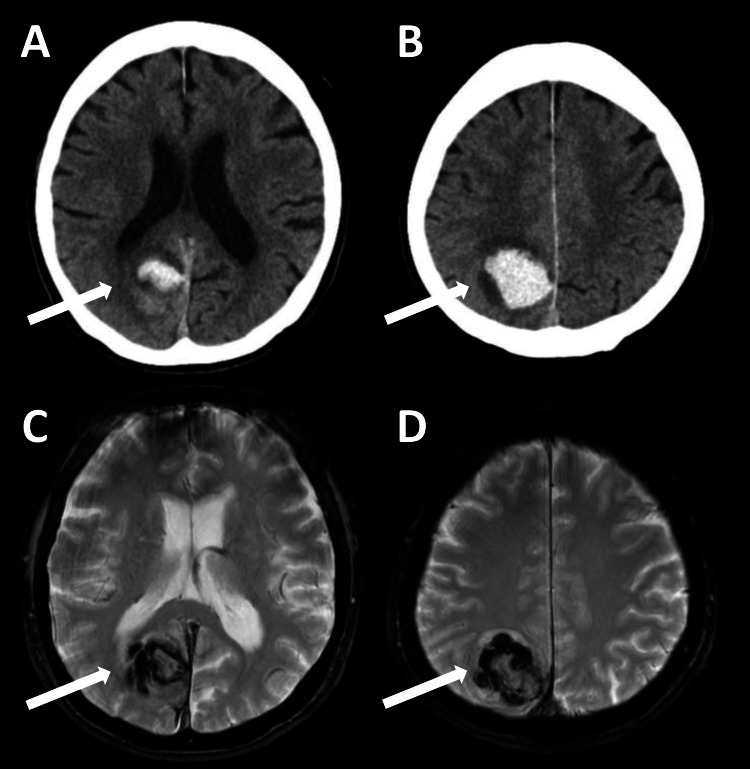
CT and MRI of the brain upon presentation. CT (A, B) and T2*-weighted MRI (C, D) of the brain obtained upon presentation demonstrating a subcortical hemorrhage in the right occipitoparietal region, accompanied by surrounding edema.

A neurological examination performed upon presentation revealed left homonymous hemianopia, left hemispheric neglect, and mild, incomplete right hemiparesis. CT and MRI of the brain revealed a subcortical hemorrhage in the right occipitoparietal lobe, with a maximum diameter of 35 mm (Figures 1A-1D). Conservative management with blood pressure control was initiated, and both the clinical symptoms and imaging findings gradually improved.

Immediately after this hemorrhagic event, the patient’s visual hallucinations completely disappeared, and his assessments were as follows: Movement Disorder Society-Unified Parkinson’s Disease Rating Scale Part III, 24; Japanese version of the Mini-Mental State Examination, 26; Frontal Assessment Battery, 12; and Japanese version of the Montreal Cognitive Assessment, 17.

**Figure 2:**
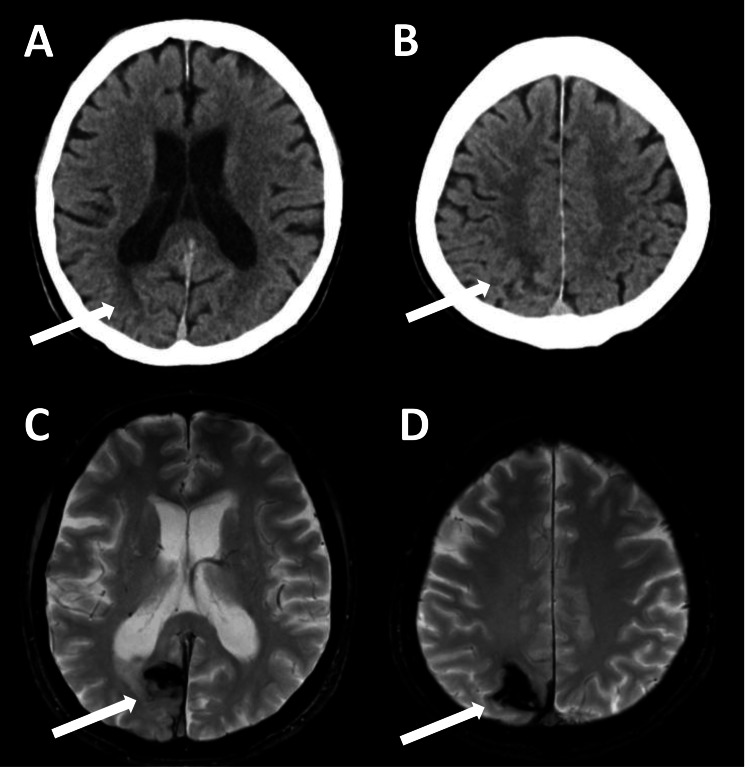
CT and MRI of the brain three months after the hemorrhagic event. CT (A, B) and T2*-weighted MRI (C, D) obtained three months after onset showing resolution of the hemorrhage and surrounding edema. The acute hematoma disappeared (A, B), and hemosiderin deposition was confined to the right precuneus (C, D).

**Figure 3:**
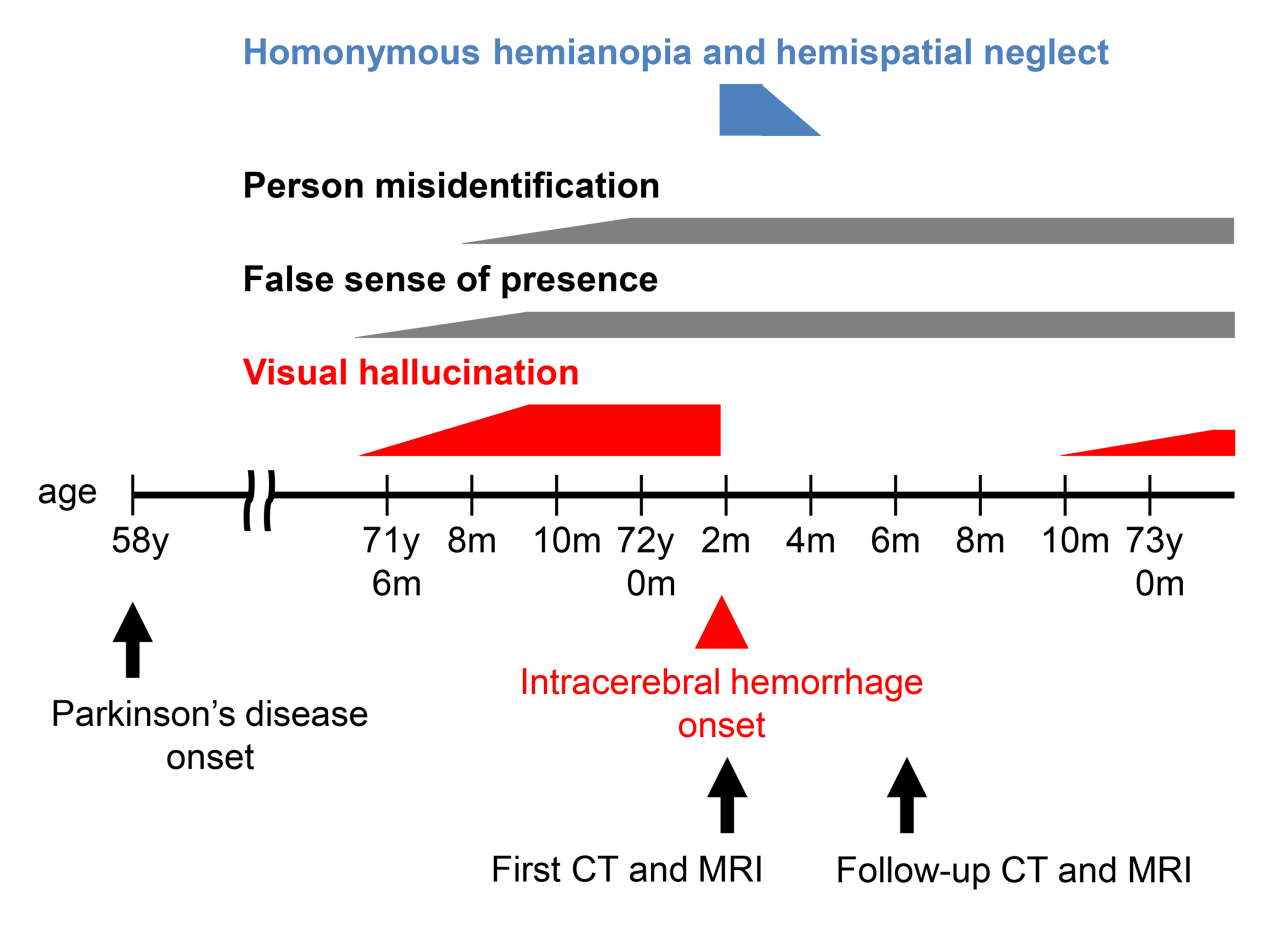
Clinical course of the patient.

Follow-up brain CT and MRI obtained three months after the hemorrhagic event showed that the residual lesion was largely confined to the right precuneus (Figures 2A-2D). At that time, the patient’s hemispatial neglect had resolved, and no hemianopia or visual hallucinations were observed. Although he occasionally experienced a false sense of presence, he did not visually perceive these experiences, while the misidentification of his wife persisted. The patient remained free of visual hallucinations for a period; however, they recurred eight months after the intracerebral hemorrhage (Figure 3). Dopaminergic and cognitive medications were not changed around the time of the hemorrhagic event.

## Discussion

In the present case, the patient’s visual hallucinations disappeared immediately after the onset of the right occipitoparietal subcortical hemorrhage. To our knowledge, this is the first reported case of a patient with PD in whom visual hallucinations resolved following a cerebrovascular hemorrhagic event.

Although homonymous hemianopia and hemispatial neglect could influence hallucinations, they do not fully explain the complete disappearance of visual hallucinations in this case, because the patient remained free of visual hallucinations even after both deficits improved.

Previous studies have reported atrophy of the cuneus, lingual gyrus, fusiform gyrus, medial occipital cortex, and inferior parietal lobule in patients with PD who experienced visual hallucinations, suggesting that impairment of the visual information processing network connecting these regions contributes to the development of these hallucinations [6]. The right occipitotemporal junction, known as the lateral occipital complex, plays an important role in shape recognition [9].

The involvement of the occipital-centered network in the generation of hallucinations is supported by the frequent occurrence of hallucinations following damage to the occipital lobe or its adjacent regions. Hallucinations have previously been reported in 9 of 128 patients (7%) with infarctions in the occipital, occipitoparietal, or occipitotemporal regions [10] and in 5 of 211 patients with lesions involving the visual cortex [11].

The present case suggests that the right precuneus may play an important role in the generation of visual hallucinations, as a follow-up MRI demonstrated a residual lesion confined to the right precuneus when visual hallucinations remained absent. Lesions involving the visual cortex itself have been reported to induce visual hallucinations. In contrast, the core lesion in our patient was centered in the precuneus, outside the primary visual cortex, and was associated with suppression of visual hallucinations.

A limitation of this report is that hallucinations were assessed based on clinical interviews and caregiver reports rather than with standardized hallucination- or psychosis-specific assessment tools.

## Conclusions

This case suggests that the right occipitoparietal cortex, particularly the precuneus, may play a critical role in the generation of visual hallucinations in right-handed patients with PD. In contrast, the persistence of a false sense of presence and person misidentification despite resolution of visual hallucinations suggests that these non-visual phenomena and delusional symptoms may arise from partially distinct neural substrates. These hypotheses, drawn from a single case, should be examined in future lesion-based and functional studies.
